# Influence of Interface on Mechanical Behavior of Al-B_4_C/Al Laminated Composites under Quasi-Static and Impact Loading

**DOI:** 10.3390/ma16216847

**Published:** 2023-10-25

**Authors:** Runwei Zhang, Zhenlong Chao, Longtao Jiang, Huimin Han, Bingzhuo Han, Shanqi Du, Tian Luo, Guoqin Chen, Yong Mei, Gaohui Wu

**Affiliations:** 1 School of Materials Science and Engineering, Harbin Institute of Technology, Harbin 150001, China; zrw920127@163.com (R.Z.);; 2 Institute of Defense Engineering, Academy of Military Science of the Chinese People’s Liberation Army, Beijing 100036, China; meiyong1990@126.com

**Keywords:** Al-B_4_C/Al laminated composites, hot pressing, rolling, interface bonding, coordinate deformation

## Abstract

In this study, Al-B_4_C/Al laminated composites with high interlayer bonding strength were fabricated by integrated hot-pressed sintering accompanied with hot rolling. The mechanical properties and interface behavior of the Al-B_4_C/Al laminated composites were investigated under quasi-static and impact loading. The results show that the Al-B_4_C/Al laminated composites obtain a high interface bonding strength, because no interlayer delamination occurs even after fractures under quasi-static and impact loads. The Al-B_4_C/Al laminated composites exhibit a better comprehensive mechanical performance, and the fracture can be delayed due to the high bonding strength interface. Moreover, laminated composites can absorb more impact energy than the monolithic material under impact loading due to the stress transition and relaxation.

## 1. Introduction

The overall performance of laminated composites is obtained by combining different elemental materials with excellent properties based on sufficient interlayered bonding. Therefore, high bonding strength is the most important purpose of preparing metal laminated composites. For the past few years, metal laminated composites, such as steel–Al [1], Al–Al [2,3], Al–Cu [4], Ti–Al [5], Cu–Zn [6], Al–Mg–Al [7], have gained extensive attention and showed great potential for replacing monolithic materials. The interlayer bonding of metal laminated composites is realized mainly by several methods, such as rolling bonding [8,9], diffusion bonding [10,11], welding bonding [12,13], and so on. However, the bonding strength is still at a limited level and the interlayer delamination often occurs when it becomes fractured under different loading. Mo et al. investigated the bending fracture behavior of AA1100/AA7075 laminated metal composites; complete fracture occurred along the interface between layers [14]. Deng et al. tested the bonding strength of steel–aluminum laminates via in situ tensile-shear experiments; cracks first emerged from the weakly bonded region, then widened and converged along the bonding interface [1]. Cao et al. found debonding and delamination on the interface of Ti/Al/Ti laminated composites under tensile loading [11]. Zhang et al. revealed the fracture mechanism of laminated Al/Ti composites; delamination appeared and extended along the Al/Ti interface [9]. Therefore, new interlayer bonding patterns of laminated composites need to be proposed to further improve the bonding strength.

A novel laminated material based on particle reinforced metal matrix composites (PRMMCs) have been developed as an ideal candidate for laminated metal materials due to low density, high specific stiffness, and strength [15,16,17,18]. Simultaneously, the laminated PRMMCs are proved to be the ideal selection for studying the different properties of laminated composites due to their excellent designability and improved interlayer bonding strength. Some preparation methods, such as spark plasma sintering [19], pressure infiltration [20], stir casting [21], and hot-pressing sintering [22], are used to produce laminated PRMMCs. Wu et al. synthesized the AA7075-AA7075/B_4_C bilayer composite via plasma activated sintering and found no interfacial cracks or delamination were observed under bend testing conditions [23]. Excellent bonding between the different layers of functionally graded AA7075-B_4_C composite with multi-layer gradient structure was also obtained [24,25]. Furthermore, some studies mainly focusing on the ballistic performance of laminated PRMMCs are reported. Karamis et al. investigated the ballistic performance of laminar metal matrix composites fabricated via squeeze casting, and observed delamination at the exit of the hole [26]. Chao et al. studied the ballistic performance of B_4_C/AA2024 functionally graded composites with wide range B_4_C volume fraction, and found that crack deflection appears in the interface of the top and middle layer [20]. A three-layered Al/7075-B_4_C/Al composite was prepared by semi continuous casting and hot rolling, significantly reducing the bullet speed during the projectile impact test [27]. It is necessary to do the basic research on the properties and mechanism of laminated PRMMCs under different loading to support more application research.

Although there have been some successful attempts to fabricate multi-layered composites and investigate their mechanical properties, a few issues still need to be paid more attention. Particularly, (i) the bonding pattern of laminated PRMMCs should be analyzed, and the interface bonding strength should be characterized. (ii) The interface behavior and fracture mechanism of laminated composites under different loadings are not systematically studied yet.

Aiming at resolving the issues above, boron carbide (B_4_C) particles-reinforced Al matrix composite (B_4_C/Al) is selected as the material system in our work to systematically study the properties of Al-B4C/Al laminated composites. B_4_C, with its light weight and high hardness, exhibits high mechanical properties at elevated temperature, chemical stability, and neutron absorption ability [28,29,30]. And 6061 aluminum alloy (6061Al) is taken as the most used and suitable metal matrix, due to its high specific strength and ductility. The Al-B_4_C/Al laminated composites are synthesized by integrated powder metallurgy in the atmosphere, which can reduce energy consumption and costs dramatically. The interface behavior of Al-B_4_C/Al laminated composites under different loadings is explored, and the fracture mechanism during a bend and impact fracture is revealed.

## 2. Materials and Methods

### 2.1. Preparation of Al-B_4_C/Al Laminated Composites

**Figure 1:**
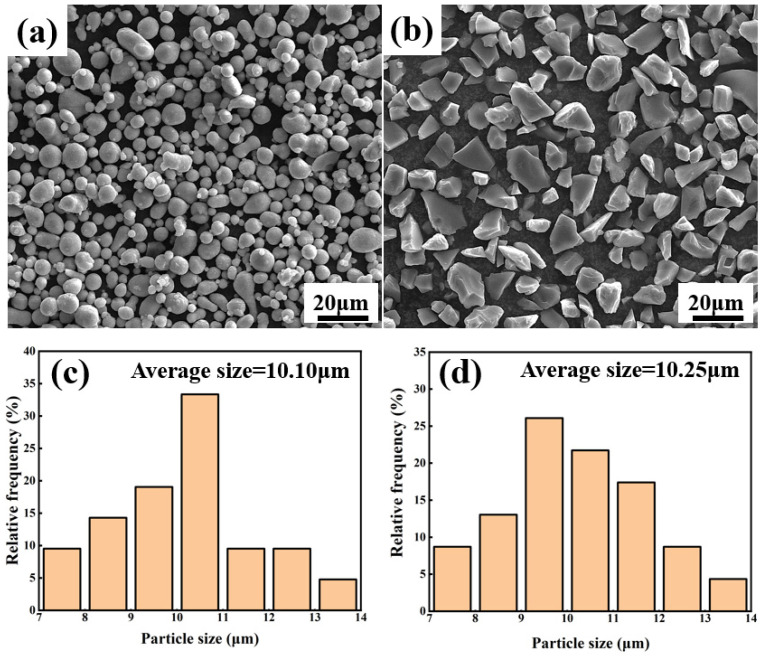
SEM photographs of selected powders: (**a**) 6061Al powders and (**b**) B_4_C powders; the scattering data of (**c**) 6061Al powders and (**d**) B_4_C powders.

6061Al powders (99.9%, D_50_ = 10 µm, Harbin Northeast Light Alloy Co., Ltd., Harbin, China) and B_4_C powders (99.9%, D_50_ = 10 µm, Dalian Jinma Boron Technology Group Co., Ltd., Dalian, China) were selected to prepare Al-B_4_C/Al laminated composites, and the SEM photographs of 6061Al powders and B_4_C powders are shown in Figure 1. It was discovered that the morphology of 6061Al powders presents an elliptical shape, and the geometry of B_4_C powders is an irregular polyhedron with sharp corners. The scattering data of powders were analyzed and given in Figure 1c,d. The average size of 6061Al powders and B_4_C powders are 10.10 µm and 10.25 µm, respectively.

**Figure 2:**
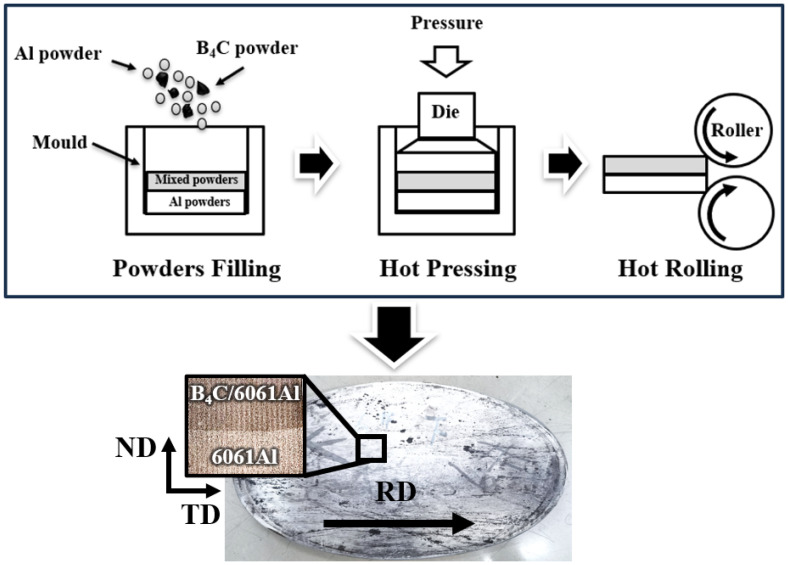
Integrated preparation process of laminated Al-B_4_C/Al composites.

The Al-B_4_C/Al laminated composites were prepared by integrated hot-pressed sintering combined with hot rolling. The schematic of the fabrication process is depicted in Figure 2. First of all, the 6061Al powders and B_4_C powders were mixed using a V-shaped powder blender at a rotating speed of 200 rpm for 4 h. Then, the 6061Al powders and the well-mixed powders were filled into a steel mold layer by layer in the order of first 6061Al and then B_4_C/6061Al, and cold pressed to form a compact powder billet. Subsequently, the steel mold filled with powders was put into a furnace and heated to 600 °C (heating rate 5 °C/min) for 3 h. The powder billet was hot pressed in the atmosphere at a pressure of 70 MPa to obtain a hot-pressed billet with a thickness of 40 mm. To increase the relative density, the hot-pressed billet was rolled for multi-passes under 450 °C, and the whole rolling reduction was 50%. The Al-B_4_C/Al laminated composites with a thickness of 20 mm were obtained after multi-pass hot rolling. In addition, solution treating (530 °C/1 h; water quenched) and aging (175 °C/6 h) were performed to further improve the strength of the Al-B_4_C/Al laminated composites.

### 2.2. Microstructure Characterization

Characterized specimens were prepared along the transverse-normal direction (TD-ND) planes (the observed section is shown in the inset of Figure 2), the interface is clad by a 15 vol.% B_4_C/6061Al layer and 6061Al layer. A scanning electron microscope (SEM, Zeiss SUPRA55, Germany) equipped with Energy Dispersive X-ray spectroscopy (EDX) was used to explore the interfacial microstructure between the different layers, and the grain morphology and fracture mechanism of the laminated composites.

### 2.3. Mechanical Properties

**Figure 3:**
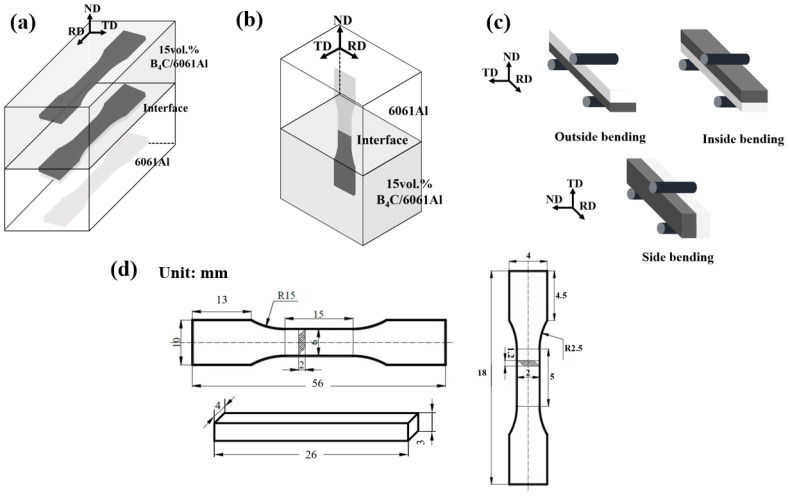
Schematics of samples under different quasi-static loading: (**a**) the RD tensile samples; (**b**) the ND tensile samples; (**c**) the three-point bending samples; (**d**) the dimensions of all samples.

The RD (rolling direction) tensile tests were performed to characterize the mechanical properties and interface performance under tensile loading. The sampling positions of the monolithic and laminated specimens for the RD tensile testing are shown in Figure 3a. Moreover, the tensile test along the normal direction (ND) was carried out in order to explore the interfacial bonding strength of the laminated composites. The sampling position is shown in Figure 3b. The section size of the ND tensile specimen is 2 mm× 1.2 mm (RD-TD), and the gauge length is 5 mm. The RD and ND tensile tests were both performed using an Instron 5569 universal testing machine (Grove City, OH, USA) equipped with an optical extensometer, and the loading speed was 0.5 mm/min.

The three-point bending tests were performed to investigate the bending properties and interface performance of the Al-B_4_C/Al laminated composites under a quasi-static bending load, and the sectioning positions of the bending samples are the same as the RD tensile samples. The three-point bending tests were also performed using an Instron 5569 universal testing machine (Grove City, OH, USA), the dimension of the bending test sample is 3 mm× 4 mm× 26 mm (width b = 4 mm, thickness d = 3 mm, bending span L = 20 mm) and the loading speed is 0.5 mm/min. The bending stress–displacement curves of the different samples are obtained by recording the variation in the bending stress (σ = 3FL/2bd^2^ (MPa), where F is the bending force) and displacement (the distance that the indenter moves down, d (mm)) until the samples fracture. Particularly, the bending tests of the laminated specimens were carried out in three forms, namely, inside bending, outside bending, and side bending. Inside bending is defined as when the B_4_C/Al composite layer of the laminated specimen is taken as the upper layer, which is subjected to compressive stress. On the contrary, outside bending means the B_4_C/Al composites layer is taken as the bottom layer, which is subjected to tensile stress. Side bending means that the bend loading force is applied on the RD-ND plane along the TD, and the press intender is perpendicular to the interface between the 6061Al layer and 15 vol.% B_4_C/Al layer. The schematic diagrams of the inside bending, outside bending, and side bending tests are shown in Figure 3c. The monolithic 6061Al, 7.5 vol.% and 15 vol.% B_4_C/Al samples were prepared and tested for comparison.

**Figure 4:**
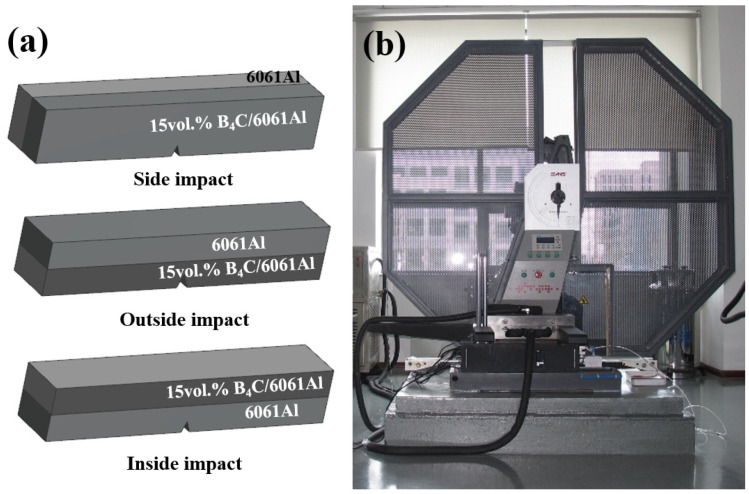
Details for Charpy impact tests: (**a**) the schematic diagram of different laminated impact samples; (**b**) the Charpy pendulum impact tester.

Charpy impact tests of the different laminated samples with a V-notch were performed to determine the impact toughness under impact loading. The dimension of the laminated samples is 55 × 10 × 10 mm^3^ with a notch depth of 2 mm (GB/T21143-2014), the schematic diagrams of the different impact samples are shown in Figure 4a. A Charpy impact testing machine ZBC2452 (Shenzhen, China) was used to measure the total absorbed energy and impact toughness (Figure 4b). The impact samples under each loading form were examined 5 times, then the results were averaged.

## 3. Results and Discussion

### 3.1. Interfacial Microstructure of Al-B_4_C/Al Laminated Composites

**Figure 5:**
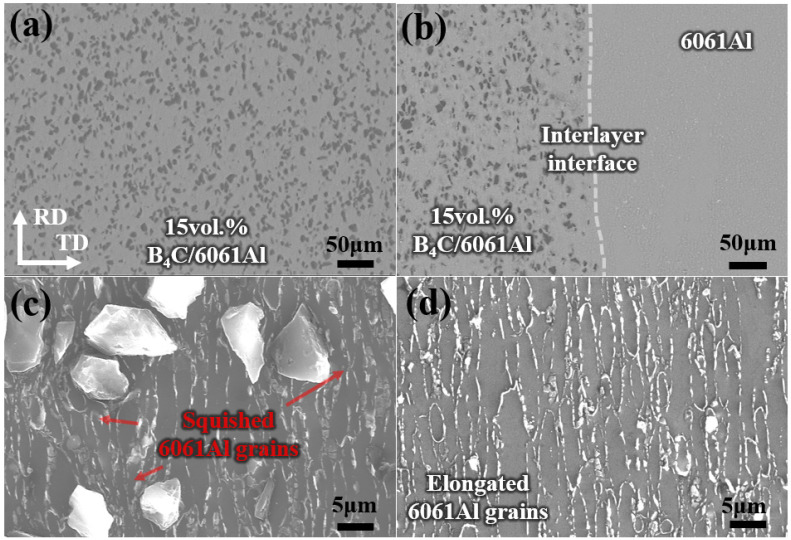
Characterization of Al-B_4_C/Al laminated composites: (**a**) microstructure of 15 vol.% B_4_C/6061Al layer; (**b**) SEM microstructure of interface between B_4_C/6061Al layer and 6061Al layer; (**c**,**d**) SEM images of 15 vol.% B_4_C/6061Al and 6061Al with etched surfaces.

The microstructure of the Al-B_4_C/Al laminated composites is given in Figure 5. The B_4_C particles exhibits a uniform distribution in the 15 vol.% B_4_C/6061Al layer, and there are no obvious agglomerations of the B_4_C particles nor voids (Figure 5a). The Al-B_4_C/Al laminated composites prepared by the integrated sintering method can obtain a high density, such as 99.7% and 99.4% for the 6061Al layer and the 15 vol.% B_4_C/6061Al layer, respectively. The interface between the different layers is clear and visible from a macro perspective of the Al-B_4_C/Al laminated composites section shown in Figure 2 (based on the distinct darkness difference between 6061Al and 15 vol.% B_4_C/6061Al). However, the interface between the B_4_C/Al layer and Al layer is not integrated nor continuous from a micro perspective in Figure 5b, which exhibits an alternative arrangement of the B_4_C particles and Al matrix [23,27,31]. The interface morphology is different from the same straight and continuous interfaces of the metal laminated composites [1,9,11,14]. Therefore, a well-bonded interface is obtained because no delamination occurs at the interface between the monolithic layers after hot rolling. Moreover, the microstructure of the 15 vol.% B_4_C/6061Al and 6061Al with the etched surface is characterized to illustrate the grain morphology in Figure 5c,d. Elongated grains are observed in both the 15 vol.% B_4_C/6061Al layer and the 6061Al layer after hot rolling, and the grain size in the 15 vol.% B_4_C/6061Al layer is squished and distorted due to the existence of the B_4_C particles. It has been demonstrated that the hot deformation of a metal matrix can be obstructed by particle reinforcements [29,32,33].

**Figure 6:**
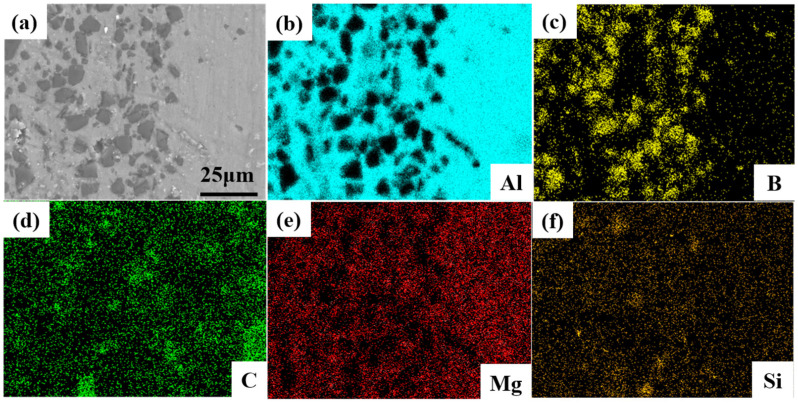
EDS analysis of the interface between B_4_C/6061Al layer and 6061Al layer at various elements: (**a**) interface SEM image; (**b**) Al; (**c**) B; (**d**) C; (**e**) Mg; (**f**) Si.

The element distribution at the interface area is analyzed in order to reveal the bonding mode between the B_4_C/6061Al layer and 6061Al layer, as shown in Figure 6. The EDS plane analysis indicates that no interface reaction layer or interlayer element diffusion layer are observed, due to the independent distribution of the Al and B elements corresponding to the B_4_C particles and Al matrix, respectively. It is indicated that the interfacial bonding of the Al-B_4_C/Al laminated composites is not achieved by an interlayer reaction [1,7,34] or element diffusion [9,11]. Instead, it is an adhesive bonding mode depending on the Al matrix to connect the B_4_C/Al layer and Al layer, and the distribution of Al matrix transits across the interlayer smoothly.

### 3.2. Mechanical Properties of Al-B_4_C/Al Laminated Composites under Quasi-Static Loading

#### 3.2.1. Tensile Properties of Al-B_4_C/Al Laminated Composites along RD

**Figure 7:**
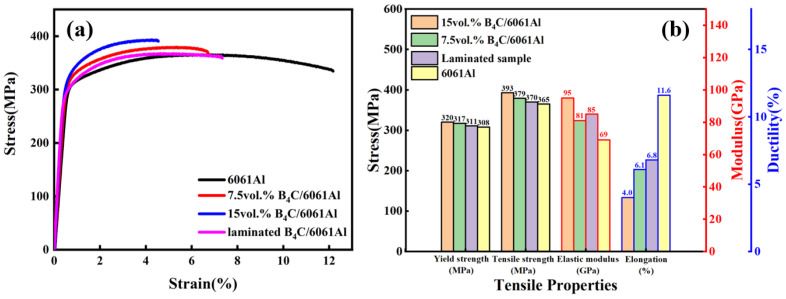
RD tensile testing results: (**a**) stress–strain curves and (**b**) mechanical properties of homogeneous and laminated composites specimen.

The engineering stress–strain curves and tensile properties of the monolithic and laminated samples along the RD are displayed in Figure 7. The strength of the monolithic samples increases with the increase in the B_4_C volume fraction, but the plasticity decreases [29,30,35,36]. The tensile strength of the 15 vol.% B_4_C/6061Al reaches 393 MPa, but the elongation is only 4%. The tensile strength of the 6061Al falls to 365 MPa, but the elongation increases to 11.6%. Additionally, the laminated B_4_C/6061Al sample obtains a poorer tensile strength compared with the 7.5 vol.% B_4_C/6061Al, but it is slightly higher than that of the 6061Al. The ductility of the laminated B_4_C/6061Al sample exbibits a contrary tendency, which is superior to the 7.5 vol.% B_4_C/6061Al and inferior to the 6061Al. Moreover, the elastic modulus of the laminated sample is slightly higher than that of the 7.5 vol.% B_4_C/6061Al due to the existence of the 15 vol.% B_4_C/6061Al layer.

**Figure 8:**
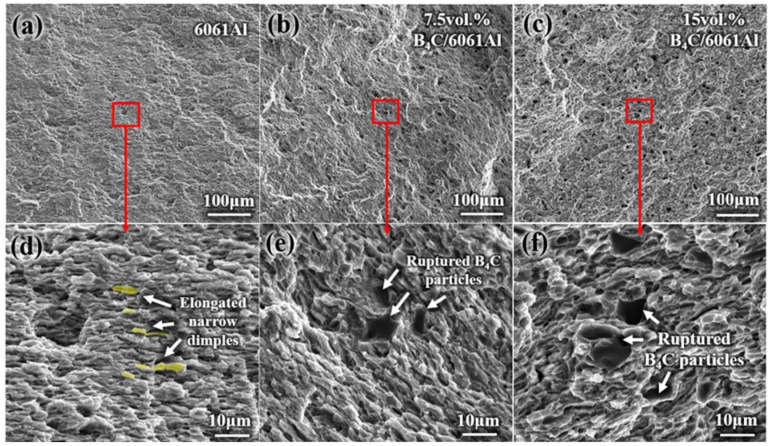
Fracture morphology of monolithic tensile samples (**a**–**c**): 6061Al, 7.5 vol.% and 15 vol.% B_4_C/6061Al; (**d**–**f**): enlarged view of red square marked in (**a**–**c**).

The SEM fracture morphology of the monolithic RD tensile samples is shown in Figure 8. Elongated narrow dimples form on the fracture in the 6061Al, because the 6061Al gains and micro voids are elongated and squished in the process of hot rolling, as shown in Figure 8d–f. Ruptured B_4_C particles and 6061Al dimples are observed in both the 7.5 vol.% and 15 vol.% B_4_C/6061Al composites as well as elongated narrow dimples, as shown in Figure 8e,f. The strong bonding between the B_4_C particles and 6061Al matrix is realized, because the ruptured B_4_C particles are still embedded in the 6061Al matrix rather than being pulled out. Moreover, the elongated dimples tend to be equiaxed with the increase in the B_4_C particle content, because the B_4_C particles prevent the rolling deformation of the 6061Al matrix [37,38].

**Figure 9:**
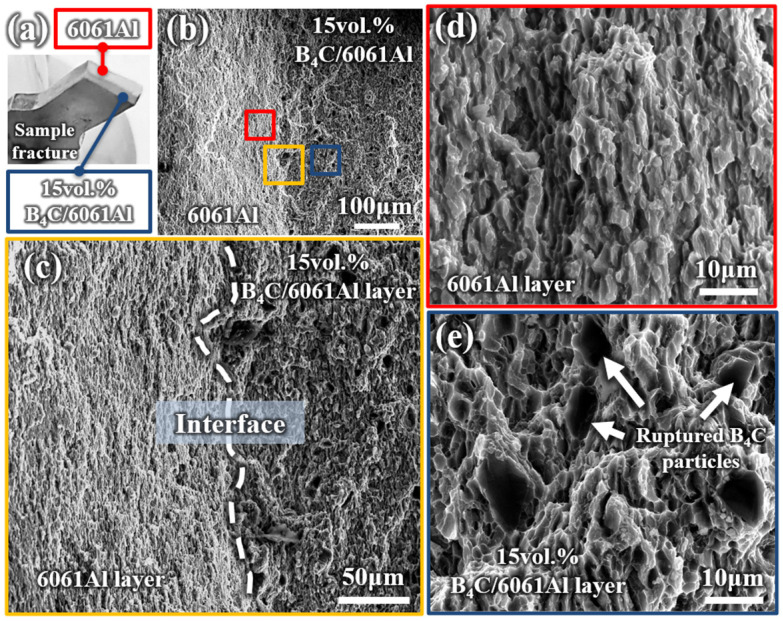
The fracture morphology (TD-ND) plane of Al-B_4_C/Al laminated tensile sample: (**a**) fractured sample; (**b**) hole fracture; (**c**) interface area of fracture enlarged view of the area marked in (**b**); (**d**,**e**) the morphology of 6061Al layer and 15 vol.% B_4_C/6061Al layer.

Figure 9 shows the TD-ND plane fracture morphology of the Al-B_4_C/Al laminated sample after tensile testing. No macroscopical interlayer crack is observed on the laminated sample fracture, as shown in the inset of Figure 9a. The interface between the 6061Al and 15 vol.% B_4_C/Al is still well-bonded after tensile fracture from the microscopic view, and the interlayer boundary tends to be blurry and zigzagged (Figure 9b,c). The fracture morphology of the 6061Al layer and 15 vol.% B_4_C/Al layer of the laminated sample are shown in Figure 9d,e. It was discovered that the fracture characteristics of the 6061Al layer and 15 vol.% B_4_C/Al layer is similar to the monolithic 6061Al and 15 vol.% B_4_C/Al, including the elongated dimples, ruptured B_4_C particles, and equiaxed dimples.

#### 3.2.2. Tensile Properties of Al-B_4_C/Al Laminated Composites along ND

**Figure 10:**
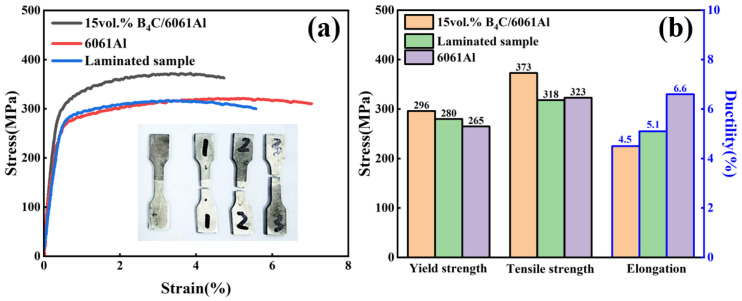
The tensile testing results of ND tensile sample: (**a**) stress–strain curves; (**b**) comparison of tensile properties.

The interfacial bonding strength of the B_4_C/Al laminated composites was tested through tensile tests along ND, and the monolithic 15 vol.% B_4_C/6061Al and 6061Al sample were also tested for comparison. The tensile stress–strain curves and mechanical properties are shown in Figure 10. The yield and tensile strength of the 15 vol.% B_4_C/Al is higher than that of the 6061Al, owing to the reinforcement of the B_4_C particles, and yet the plasticity of the 15 vol.% B_4_C/Al is less favorable. The strength of the laminated sample is close to the 6061Al matrix, and the elongation is between the B_4_C/Al composites and 6061Al. It is interesting that the fracture occurs in the 6061Al part of the ND tensile laminated sample instead of the interface between the 15 vol.% B_4_C/Al and 6061Al (the inset of Figure 10a). It is qualitatively concluded that the interfacial bonding strength is higher than the tensile strength of the 6061Al. Such a high interface bonding strength is obtained by the adhesive bonding of the Al matrix owing to the integrated (or one-step) sintering forming. It is much higher than the bonding strength of the steel–aluminum laminates (tensile-shear testing) [1] and Ti/Al/Ti laminated composites (T-peeling testing) [5,11], which are usually prepared by a two-step method (reaction bonding, diffusion bonding, and mechanical bonding) [7,9,34].

**Figure 11:**
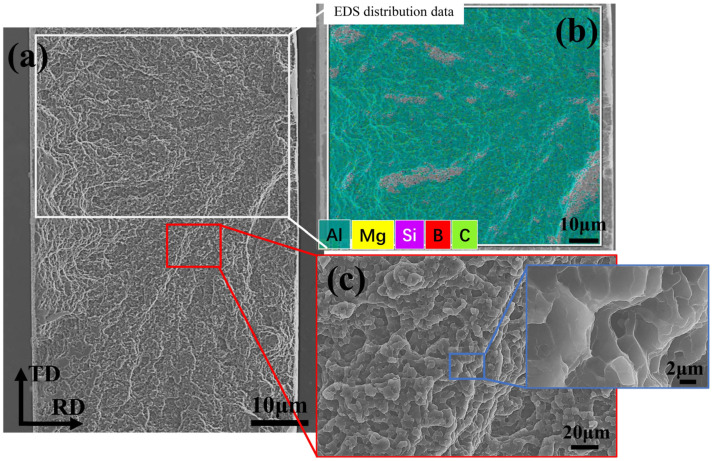
The fracture and the EDS analysis of Al-B_4_C/Al laminated sample: (**a**) morphology of the hole fracture; (**b**) EDS map scanning of the white rectangular area marked in (**a**); (**c**): enlarged view of fracture.

The fracture analysis of the Al-B_4_C/Al laminated sample obtained after the ND tensile testing is shown in Figure 11. It is determined to be a fracture in the 6061Al part, because no B_4_C particles appear in the hole fracture of the laminated sample as shown in Figure 11a. The element composition of the fracture consists of an overwhelming majority being Al and a minority being Mg and Si, and neither the B nor C elements are detected, according to the EDS surface analysis in Figure 11b. This phenomenon is different from the previous research on interface debonding. For instance, Cao et al. found that Al falls off the Al matrix and adheres to the Ti surface during the peeling tests when the fracture occurs on the interface [11]. Deng et al. also reported that the residual aluminum matrix on the steel side appears as a discontinuous narrow strip, perpendicular to the tensile direction in the steel-side shear fracture [1]. The fracture morphology exhibited a typical ductile fracture with dimples, as shown in Figure 11c. Moreover, it is worth mentioning that the strength of the RD tensile samples is generally higher than that of the ND tensile samples, because the hot rolling can enhance the strength of the composites along the RD [3,22,37,39].

#### 3.2.3. Bending Properties of Al-B_4_C/Al Laminated Composites

**Figure 12:**
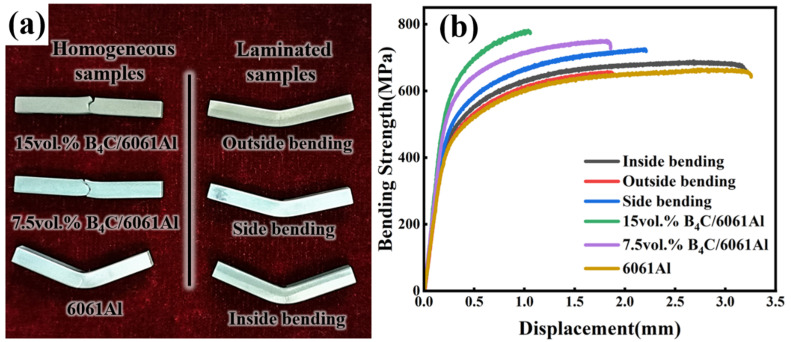
The bending test of monolithic and laminated samples: (**a**) photos of monolithic and laminated samples after bending testing; (**b**) bending strength–displacement curves of different samples.

The bending properties of the monolithic and Al-B_4_C/Al laminated samples are characterized, and the results are given in Figure 12 and Table 1. Figure 12a shows the photographs of the monolithic and laminated samples after the bending tests. The 15 vol.% and 7.5 vol.% B_4_C/6061Al composite samples rupture completely with less deformation, while the 6061Al sample fractures due to a large bending deformation, but is not broken completely. The bending strength increases from 662MPa to 780MPa with the volume fraction of B_4_C particles increasing from 0 to 15 vol.%, but the bending fracture displacement decreases with the increase in the B_4_C particle content, as listed in Table 1.

Moreover, all forms of the Al-B_4_C/Al laminated samples cracked during the bending process, but none of them fractured completely. Among them, the outside bending laminated sample fractured first with a fracture displacement of 1.9 mm, which is comparable to that of the 7.5 vol.% B_4_C/6061Al. However, the fracture displacements caused by inside and side bending the laminated samples can reach 3.2 mm and 2.2 mm (much higher than that of the 7.5 vol.% B_4_C/6061Al), which means the fracture can be delayed significantly. The strength–displacement curves (Figure 12b) indicate the bending strength of all forms of Al-B_4_C/Al laminated samples are lower than that of the 7.5 vol.% B_4_C/6061Al, but higher than that of the 6061Al until fracture. The side bending sample exhibits the highest bending strength, at 723 MPa, while the outside bending sample obtains the lowest bending strength, at 655 MPa (but higher than the 6061Al at the same displacement).

**Figure 13:**
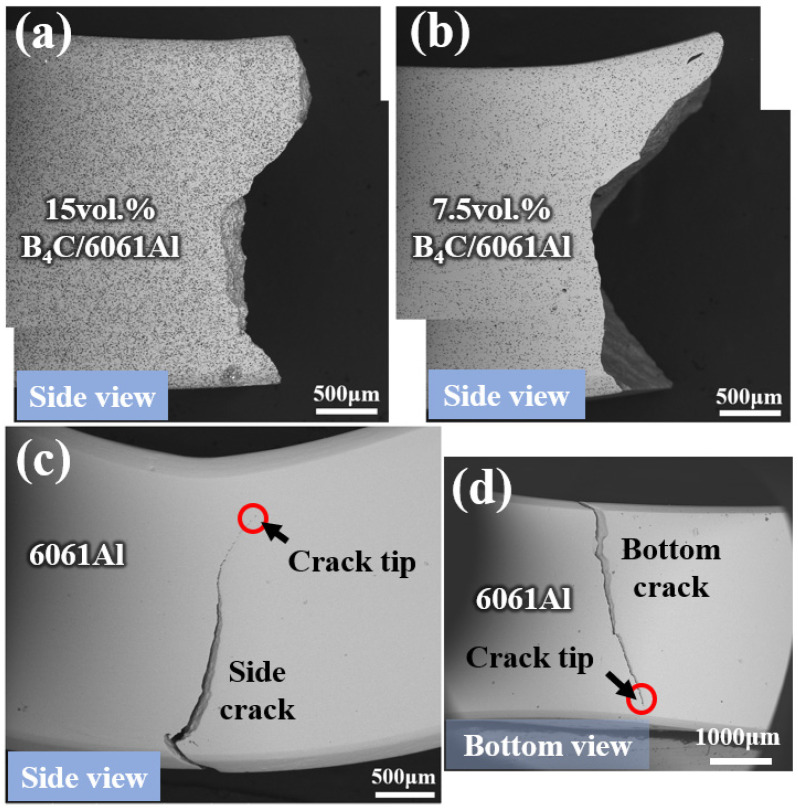
The fracture morphology of monolithic bending samples: (**a**) 15 vol.% B_4_C/6061Al; (**b**) 7.5 vol.% B_4_C/6061Al; (**c**) 6061Al from side view and (**d**) 6061Al from bottom view.

Figure 13 represents the crack morphology of the different monolithic samples from different views. The fracture of the 15 vol.% B_4_C/6061Al sample is relatively flat, while the 7.5 vol.% B_4_C/6061Al sample obtains a jagged fracture appearance (Figure 13a,b). That means more deformation occurs to the 7.5 vol.% B_4_C/6061Al until fracture occurs. A large crack emerges in the 6061Al sample, but does not crack through the whole sample from both the side and bottom view (Figure 13c,d). It is concluded that the fracture mode transforms from a brittle fracture to a ductile fracture with the decrease in the B_4_C content.

**Figure 14:**
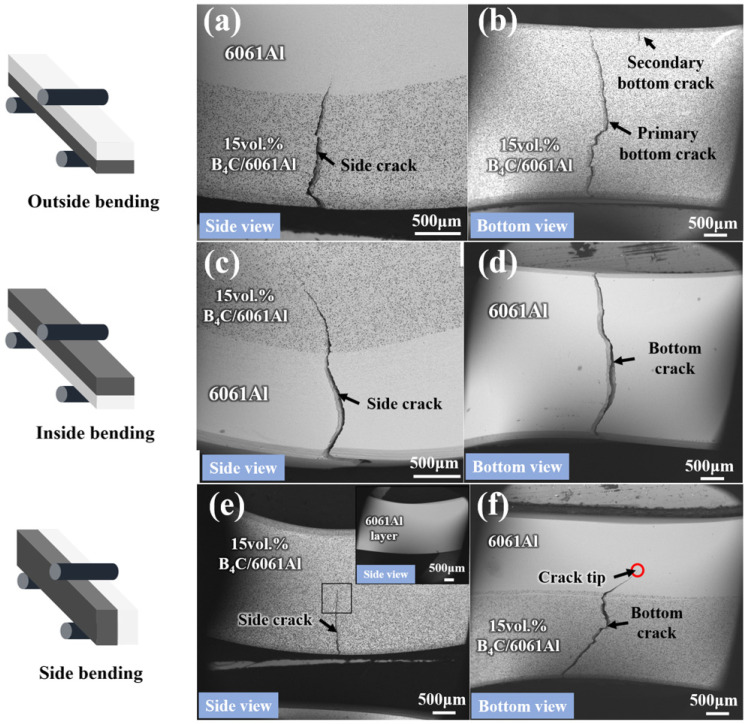
The fracture morphology of Al-B_4_C/Al laminated bending samples from side and bottom view: (**a**,**b**) outside bending sample; (**c**,**d**) inside bending sample; (**e**,**f**) side bending sample.

Figure 14 shows the fracture morphology of the outside, inside, and side Al-B_4_C/Al laminated bending samples from different views. Observed in the fracture of the outside bending sample (Figure 14a,b), a large side crack initiates from the 15 vol.% B_4_C/6061Al layer and stops in the 6061Al layer; the crack propagation path is flexural. A large primary crack as well as a thin secondary bottom crack occurs in the 15 vol.% B_4_C/6061Al layer from the bottom view (Figure 14b), and the primary crack propagates through the whole sample. In reverse, the side crack initiates from the 6061Al layer of the inside bending sample, and terminates in the 15 vol.% B4C/6061Al layer observed from Figure 14c,d. The bottom crack runs through the 6061Al bottom layer, but without secondary cracks (Figure 14d). Figure 14e,f show the asymmetry fracture morphology of the side bending laminated sample from the side and bottom views. The side crack appears in the 15 vol.% B_4_C/6061Al layer (Figure 14e), but does not appear in the 6061Al layer (inset of Figure 14e). The bottom crack initiates from the 15 vol.% B_4_C/6061Al layer and terminates in the 6061Al layer, as shown in Figure 14f. The bottom crack exhibits a z-shaped propagation after being deflected twice at large angles, without running through the whole sample.

### 3.3. Properties of Al-B_4_C/Al Laminated Composites under Impact Loading

**Figure 15:**
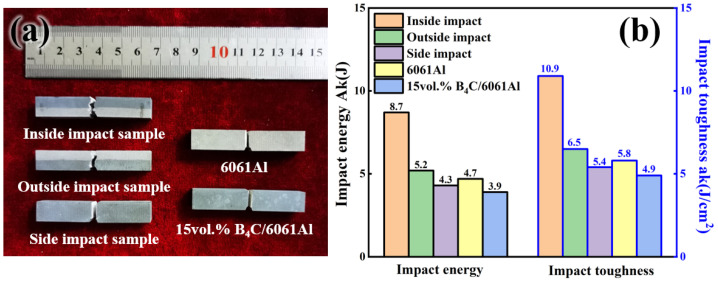
Charpy impact results of Al-B_4_C/Al laminated and monolithic bending samples: (**a**) recovered samples after impact fracture; (**b**) impact energy and impact toughness of different samples.

Figure 15 shows the Charpy impact results of the Al-B_4_C/Al laminated and monolithic bending samples. The photo of all the forms of the recovered monolithic and laminated samples are shown in Figure 15a. The fractures in the outside and inside impact samples are more irregular, while the fracture of the monolithic and side laminated samples are relatively flat. The impact toughness of the laminated samples is higher than that of the monolithic samples, except for the side impact laminated sample, as shown in Figure 15b. The inside and outside impact samples absorb relatively more impact energies of 8.7 J and 5.2 J, while the monolithic 6061Al and 15 vol.% B_4_C/6061Al samples absorb minor impact energy of 4.7 J and 3.9 J. It is concluded that the laminated configuration has a great influence on the impact toughness of the B_4_C/6061Al composites.

**Figure 16:**
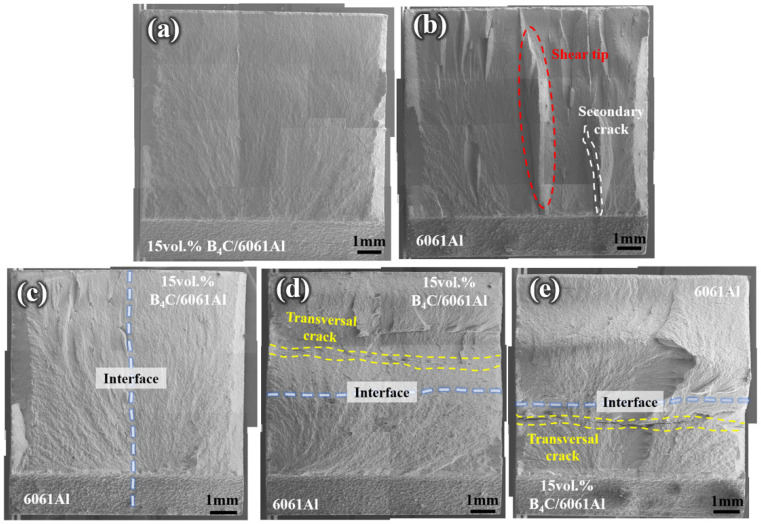
The fractographic images of monolithic samples obtained after Charpy impact testing: (**a**) 15 vol.% B_4_C/6061Al, (**b**) 6061Al, (**c**) side impact laminated sample, (**d**) outside impact laminated sample and (**e**) inside impact laminated sample.

The crack initiates at the notch edge and further propagates until the sample is fractured during the Charpy impact testing, generating the main fracture characteristic areas, including the crack initiation, propagation, and tip shearing areas [40,41]. The fractographic images of the monolithic and laminated B_4_C/6061Al samples obtained after the Charpy impact testing are shown in Figure 16. The entire fracture surface of the 15 vol.% B_4_C/6061Al sample is relatively flat, while the whole fracture surface of the 6061Al sample is waved with large shear pits and deep secondary cracks (Figure 16a,b). Therefore, the Charpy impact toughness of the 6061Al is greater than that of the 15 vol.% B_4_C/6061Al, because the appearance of the shear tips and secondary cracks leads to additional energy dissipation [42,43]. This is consistent with the impact toughness testing results presented in Figure 15.

The whole fracture surface of the side impact laminated sample consisting of 6061Al (left half) and 15 vol.% B_4_C/6061Al (right half) is shown in Figure 16c. The shallow pits occur on the 6061Al half, which are smaller than that of the fracture in the 6061Al monolithic sample. The flat fracture surface of the 15 vol.% B_4_C/6061Al half is similar to the surface of the 15 vol.% B_4_C/6061Al monolithic sample. The impact toughness of the side impact laminated sample (5.4 J/cm^2^) is between 6061Al (5.8 J/cm^2^) and 15 vol.% B_4_C/6061Al (4.9 J/cm^2^). The fractures of the inside and outside impact laminated samples are both composed of 6061Al and 15 vol.% B_4_C/6061Al, as shown in Figure 16d,e. The entire fracture surfaces of these samples exhibit peaks and valleys with large ups and downs, and transverse cracks (perpendicular to the impact direction) run through the whole crack surface. The large deformation and cracks imply the severe interaction on the fractures of the inside and outside impact laminated samples, and the maximum toughness of these samples (10.9 and 6.5 J/cm^2^) are obtained.

## 4. Discussion

### 4.1. Interface Behavior and Fracture Mechanism of Al-B_4_C/Al Laminated Composites under Quasi-Static Loading

**Figure 17:**
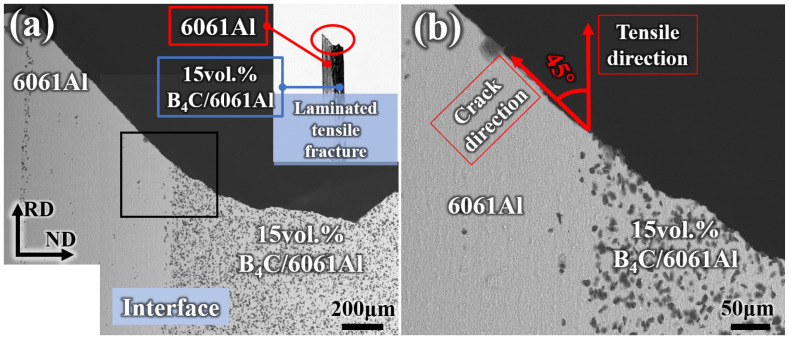
RD-ND fracture morphology of Al-B_4_C/Al laminated tensile sample: (**a**) overall fracture; (**b**) enlarged view of the area marked in (**a**).

The fracture pattern of the Al-B_4_C/Al laminated sample can be inferred from the RD-ND fracture appearance shown in Figure 17. It is further confirmed that no interlayer delamination occurs after tensile failure observed from the inset of Figure 17a. The fracture morphology of the 15 vol.% B_4_C/Al layer is overall flat but with zigzags (Figure 17a), while the 6061Al layer cracks along a 45° angle to the tensile direction (Figure 17b). The reason for this might be that the 6061Al layer undergoes a large deformation with the increasing tensile loads, and fractures first when the tensile stress exceeds its tensile strength. Then, the crack propagates through the interface, and leads to the abrupt failure of the 15 vol.% B_4_C/Al layer due to its poor plasticity. The fracture morphology of the Al-B_4_C/Al laminated composites is different from those with a flat fracture (observed from the side view), as reported by other works [11,44].

**Figure 18:**
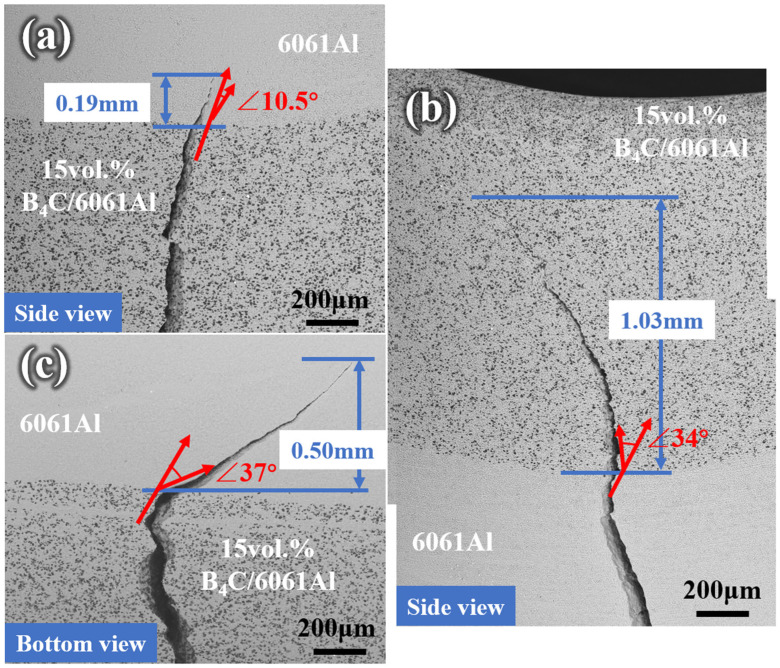
The crack propagation across the interface of Al-B_4_C/Al laminated samples obtained after three-point bending tests: (**a**) outside, (**b**) inside, and (**c**) side bending.

The crack propagation path through the interfaces of the Al-B_4_C/Al laminated composites under different forms of bending loads are shown in Figure 18. The side crack initiates from the 15 vol.% B_4_C/6061Al and grows through the interface of outside bending sample, finally terminating in the 6061Al layer (Figure 18a). The crack tip is near the interface with the shortest vertical distance of 0.19 mm, which means limited overall deformation occurs. The side crack initiating from the 6061Al layer propagates through the interface of the inside bending sample and continues to expand out, as shown in Figure 18b. It terminates near the upper edge of the 15 vol.% B_4_C/6061Al layer, which is 1.03 mm away from the interface. The large overall deformation can be ensured to delay failure based on the sufficient plasticity of the 6061Al layer and the high strength interface. The vertical distance from the interface to the crack termination point in the 6061Al layer of the side bending sample is 0.50 mm (Figure 18c), which is greater than the outside bending sample but less than the inside bending sample. Moreover, the crack can be deflected when it propagates through the interface of the Al-B_4_C/Al laminated composites [44,45]. The crack deflection angle of the side bending is 37°, which is greater than those of the inside and outside bending (34° and 10.5°, respectively). This means that the fracture of the side bending sample needs the most energy, which is consistent with the results of Figure 12.

### 4.2. Fracture of Al-B_4_C/Al Laminated Composites under Impact Loading

**Figure 19:**
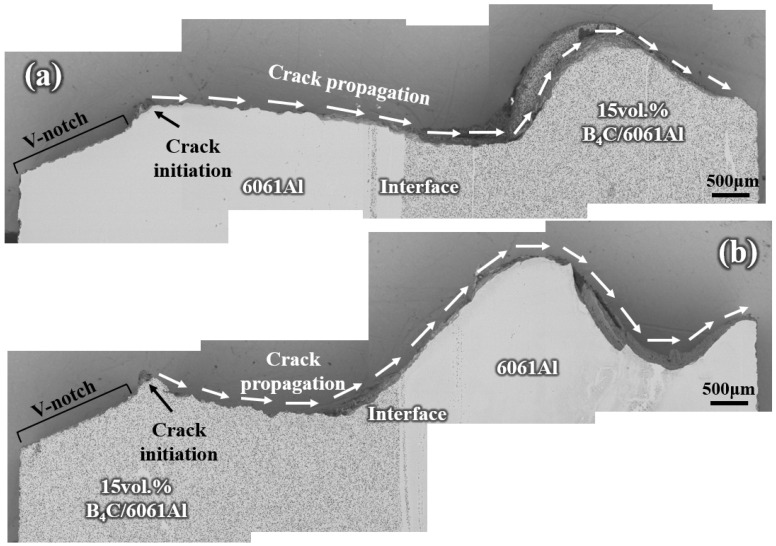
The crack propagation path of Al-B_4_C/Al laminated samples obtained after Charpy impact testing from the side view: (**a**) inside laminated sample and (**b**) outside laminated sample.

**Figure 20:**
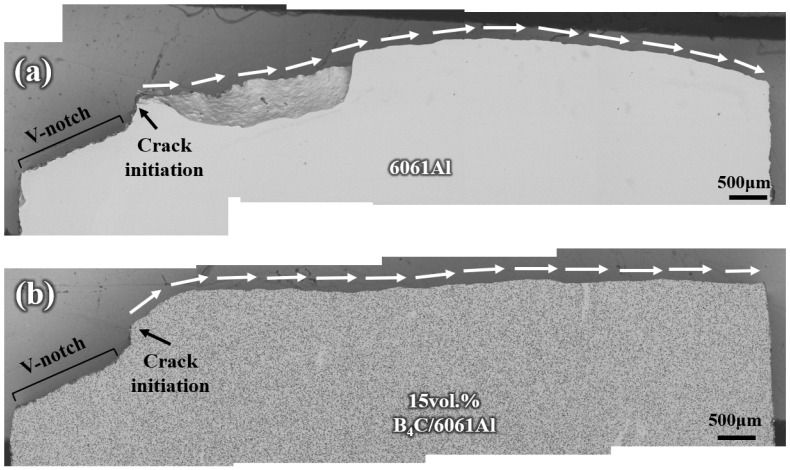
The crack propagation path of side impact sample obtained after Charpy impact testing from the side view: (**a**) 6061Al part and (**b**) 15 vol.% B_4_C/6061Al part.

The crack propagation path of the Al-B_4_C/Al laminated composites under different forms of Charpy impact loading are shown in Figure 19 and Figure 20. As shown in Figure 19a, the crack initiates from the 6061Al layer and propagates through the interface of the inside impact sample smoothly and continuously without delamination. Then, the crack propagation path suddenly waves up and down until the sample fractures completely. This indicates the stress can be effectively transmitted from the 6061Al layer to the 15 vol.% B_4_C/6061Al layer, based on the high interlayer bonding strength [46]. The unfractured part of the 15 vol.% B_4_C/6061Al layer cannot bear the stress and releases this rapidly with a large wavy crack [43]. As for the outside impact sample, the crack initiates from the 15 vol.% B_4_C/6061Al layer and leaps up before reaching the interlayer interface, as shown in Figure 19b. Although the total energy absorbed during the fracture includes the crack initiation and crack propagation energies, the crack initiation consumes the majority of the constituent energy during the impact testing [41]. The 6061Al layer of the inside impact sample is not inclined to crack due to its good plasticity, while the 15 vol.% B_4_C/6061Al layer (with poorer plasticity) of the outside impact sample cracks more easily. Moreover, the tensile stress can be transmitted steadily by the 6061Al layer until the crack propagation path suddenly deflects through a large angle after crossing the interface. Compared with the inside impact sample, the crack propagation path deflects earlier in the 15 vol.% B_4_C/6061Al layer of the outside impact sample before it reaches the interface. Less energy is dissipated because the stress is released prematurely. Therefore, the inside impact sample is more beneficial to relieve the stress concentration and absorbs more energy, compared with the outside impact sample, which is consistent with the Charpy impact results of Figure 15. Although the stress is released prematurely through a large angle deflection and fracture, it still can transmit through the interface from the 15 vol.% B_4_C/6061Al layer to the 6061Al layer without delamination.

However, the propagation paths of the side impact sample consist of the 6061Al part and 15 vol.% B_4_C/6061Al part, due to the material asymmetry in the direction perpendicular to the impact, as shown in Figure 20. The propagation paths of both parts are overall flat without fluctuations because of the existence of the 15 vol.% B_4_C/6061Al, compared with the outside and inside laminated samples. No significant plastic deformation occurs during the process of the impact fracture, which means there is poor impact energy absorption.

## 5. Conclusions

1. The Al-B_4_C/Al laminated composites with a high interface bonding strength are prepared through integrated hot-pressed sintering in the atmosphere.
2. The comprehensive performance of the Al-B_4_C/Al laminated composites is obtained based on the high interface bonding strength, because no interface delamination occurs even after fractures under quasi-static and dynamic loading.
3. The fracture of the Al-B_4_C/Al laminated composites can be delayed through adjusting the laminated structure form (or loading form), compared with the homogeneous B_4_C/Al composites.
4. The laminated structure of the Al-B_4_C/Al composites improves the energy absorption effect during the impact fracture, due to the stress transition and relaxation based on the high interface bonding strength.

## Figures and Tables

**Table 1:** Bending experiment results of monolithic and laminated samples.

Samples	Bending Strength (MPa)	Displace (mm)
15 vol.% B_4_C/6061Al	780	1.0
7.5 vol.% B_4_C/6061Al	750	1.9
6061Al	662	3.3
Outside bending	655	1.9
Side bending	723	2.2
Inside bending	677	3.2

## Data Availability

The research data are available on request from the corresponding author.
